# UPLC-PDA-MS/MS Profiling and Healing Activity of Polyphenol-Rich Fraction of *Alhagi maurorum* against Oral Ulcer in Rats

**DOI:** 10.3390/plants11030455

**Published:** 2022-02-07

**Authors:** Hala El-Zahar, Esther T. Menze, Heba Handoussa, Ahmed K. Osman, Mohamed El-Shazly, Nada M. Mostafa, Noha Swilam

**Affiliations:** 1 Department of Pharmaceutical Sciences, Faculty of Dentistry, British University in Egypt (BUE), Cairo 11837, Egypt; hala.elzahar@bue.edu.eg; 2 Department of Pharmacology and Toxicology, Faculty of Pharmacy, Ain Shams University, Cairo 11566, Egypt; esther.menze@gmail.com; 3 Department of Pharmaceutical Biology, Faculty of Pharmacy and Biotechnology, German University in Cairo (GUC), Cairo 11835, Egypt; heba.handoussa@guc.edu.eg; 4 Department of Botany and Microbiology, Faculty of Science, South Valley University, Qena 83523, Egypt; ahmosman2000@yahoo.com; 5 Department of Pharmacognosy, Faculty of Pharmacy, Ain Shams University, Cairo 11566, Egypt; 6 Department of Pharmacognosy, Faculty of Pharmacy, The British University in Egypt (BUE), Cairo 11837, Egypt

**Keywords:** *Alhagi maurorum*, polyphenols, LC-MS-MS, antioxidant, oral ulcer, anti-inflammatory

## Abstract

Camelthorn, *Alhagi maurorum* Boiss, family Fabaceae has long been used in African folk medicine owing to its richness in pharmacologically active metabolites. The crude extract (CEAM), ethyl acetate fraction (EFAM) and *n*-butanol (BFAM) fraction of *A. maurorum* aerial parts were investigated for their total polyphenols and oral antiulcer activity using in-vitro and in-vivo models. The major phenolic compound was isolated from the polyphenol-rich EFAM fraction and identified by conventional and spectroscopic methods of analysis as isorhamnetin-3-*O*-rutinoside. Furthermore, standardization of EAFM using UPLC-PDA-UV quantified isorhamnetin-3-*O*-rutinoside as 262.91 0.57 g/mg of the fraction. Analysis of EFAM using UPLC-PDA-MS/MS revealed tentative identification of 25 polyphenolic compounds. EFAM exhibited the most potent free radical scavenging activity against DPPH, with an IC_50_ (27.73 ± 1.85 µg/mL) and an FRAP value of (176.60 ± 5.21 μM Trolox equivalent (TE)/mg fraction) in comparison with CEAM and BFAM. Acetic acid-induced oral ulcers in a rat model were used to evaluate the healing properties of *A. maurorum* aerial parts. EFAM significantly decreased tumor necrosis factor-alpha (TNF-α) and interleukin-2 (IL-2) by 36.4% and 50.8%, respectively, in the ulcer tissues while, CEAM and BFAM exhibited lower activity at the same dose. In addition, EFAM led to a significant (*p* < 0.0001) rise in the expression of proliferating cell nuclear antigen (PCNA), a cell proliferation marker. *A. maurorum* exhibited a potent healing effect in acetic acid-induced oral ulcers in rats by mitigating the release of pro-inflammatory cytokines and improving PCNA expression.

## 1. Introduction

Oral mucosal ulcers are painful shallow sores or open lesions affecting the epithelium or the underlying connective tissues of the oral mucosa [1]. Many factors contribute to the development of oral ulcers including trauma, burns, bacterial or viral infection, drug-induced side effects or allergic reaction, stress, recurrent aphthous ulcers, Behçet’s syndrome, and oral lichen planus [1]. The buccal mucosa is the most commonly affected area of the oral cavity, followed by the tongue, and finally the lower lip. Due to the recurrent stimulation of the tissues, these lesions can persist for weeks, especially for tongue ulcers. Oral ulcers can be classified as acute or chronic ulcers based on their appearance and progression. Ulcers lasting longer than 14 days are classified as chronic [2]. Oxidative stress is strongly associated with oral ulcer progression [3]. Reactive oxygen species (ROS) are incorporated in many oral conditions [4]. Plant polyphenols possess a potent therapeutic antioxidant effect protecting tissues against oxidative damage. They suppress ROS formation by inhibiting oxidative-related enzymes, scavenging ROS, or upregulating the antioxidant defense mechanisms. They modulate the inflammatory process by interacting with ROS and terminating chain reactions even before affecting cell viability [5]. Polyphenols can promote the ulcer healing process by reducing pro-inflammatory cytokines implicated in acute inflammation, down-regulating the cellular and intercellular adhesion agents, and blocking both leukocyte-endothelium contact and inflammatory process nuclear signaling pathways [6]. Polyphenols have been found to reduce inflammation and improve the recovery process of chemically induced ulcers on rat tongues [7].

Another study found that using polyphenols improved wound healing in oral sores and periodontitis [8]. Wound healing occurs following disruption or damage to the normal anatomical structure and function and depends on the power of tissue regeneration, resulting in a coordinated series of cellular and molecular processes including hemostasis, inflammation, proliferative phase, angiogenesis, wound contracture, epithelialization, and matrix remodeling [9].These processes depend on homogenous functions of neutrophils, macrophages, fibroblasts, and endothelial cells that are controlled by the interaction with other cells, extracellular matrix proteins, and growth factors. Any change in these processes will lead to abnormal healing processes affecting successful wound healing [10]. Any drug which interferes with the formation of clots, inflammatory reactions, or cell proliferation can be used in the treatment of ulcers. The most widely consumed agents in treating oral ulcers are corticosteroids and antibiotics; however, their use may be limited owing to potential complications [11,12] Local anti-inflammatory drugs and painkillers are the first lines of treatment for patients with oral ulcers; however, long-term use may result in serious complications such as secondary fungal infections and drug resistance [13]. It has been reported that the use of natural remedies in treating oral sores can result in an improved recovery rate with minimal adverse effects [14].These findings encourage scientists to search for new effective, cheap, and safe remedies from nature for the treatment of oral ulcers.

*Alhagi maurorum* Boiss (Camelthorn) is a widely distributed folk medicinal plant that belongs to the family Fabaceae. It is a perennial, spiny, densely branched shrub, with deep roots that can extend 15 m into the ground. The plant quickly colonizes an area by producing new plants owing to its creeping roots [15]. Traditionally, the plant roots, aerial parts, and manna are used to cure different diseases in different areas especially in the MENA and Central Asian countries. In Egypt and Afghanistan, *A. maurorum* is used to treat gastrointestinal, liver, and urinary tract disorders [15]. It is traditionally recommended for treatment of progressive ulcers, hemorrhoids, cough, rheumatic pain, angina, eczema, and gastritis in Pakistan, Uzbekistan, and Saudi Arabia [16].The manna of *Alhagi* is reported in the Islamic traditional medicine (ITM) books for its laxative, detergent, and antipyretic properties, and as a cure for cough and liver bile diseases [16]. The crude extract of *A. maurorum* aerial parts exhibited a protective effect against peptic ulcers and gastroesophageal reflux in rats [17]. Another study reported antiulcerogenic activity of *A. maurorum* in treating gastric ulcers [15]. Pourahmad et al. [11] revealed that *A. maurorum* steam distillate was effective in treating recurrent aphthous ulcers. In light of the cited studies, nothing could be found in the literature exploring the metabolic profile of the *A. maurorum* aerial parts responsible for its healing potential in oral ulcers or examining the related healing mechanisms. Thus, the present study aimed at the standardization and profiling the polyphenol-rich fraction ethyl acetate (EFAM) of *A. maurorum* aerial parts by adopting the UPLC-PDA and UPLC-PDA-ESI-MS/MS techniques. In addition, the evaluation of the antioxidant activity of *A. maurorum* crude extract (CEAM) and its fractions, namely, ethyl acetate (EFAM), and *n*-butanol (BFAM), were evaluated by DPPH and FRAP assays. Furthermore, the antiulcerogenic activity of *A. maurorum* aerial parts extract and fractions was evaluated in vivo on chemically induced tongue ulcers in rats.

## 2. Results

### 2.1. Extraction and Fractionation of A. maurorum

One of the main goals of this study is to prepare a standardized polyphenol-rich fraction from the aqueous alcohol extract of the aerial parts of *A. maurorum*. This incorporates extraction, fractionation and standardization. The aqueous alcohol extract CEAM was fractionated using increasing the polarity solvents n-hexane, ethyl acetate, and n-butanol. To obtain fractions with a high concentration of polyphenols, the n-hexane fraction was prepared first as a step in the purification and extraction of relatively nonpolar components, followed by the preparation of the ethyl acetate (EFAM) and n-butanol (BFAM) fractions.

### 2.2. Determination of Total Polyphenolic and Flavonoid Content of A. maurorum

The spectrophotometric assays of the total polyphenolic and flavonoid content revealed that the highest polyphenol and flavonoid content was found in EFAM, followed by CEAM and BFAM (Table 1).

### 2.3. Identification of the Major Polyphenol Compound of EFAM

Phytochemical investigation of the EFAM led to the isolation and identification of the major flavonoid compound, isolated as a yellow powder (12.8 mg): ^1^H NMR data (400 MHz, DMSO-*d*6): aglycone: *δ*_H_ 3.83 (3H), 6.17 (d, *J* = 2.0 Hz, 1 H, H-6), 6.46 (d, *J* = 2.0 Hz, 1 H) 6.38 (1H, d, *J* = 2.0 Hz, H-8),7.85 (1H, d, *J* = 2 Hz, H-2′), 6.91 (1H, d, *J* = 8.4 Hz, H-5ʹ), 7.51 (1H, dd, *J* = 8.4, 2.0 Hz, H-6′); sugar moiety: *δ*_H_ 1.02 (3H, d, *J*=6.2 Hz, H-6″rham), 3.12-3.86 (10 H, m, other sugar protons), 4.57 d (1.4) 4.42 (1H, *br*. *s*, H-1″rham), and 5.41 (1H, d, *J* = 8.5 Hz, H-1″glc). ^13^C NMR data (100 MHz, DMSO-*d*6) 156.93 (C-2), 133.70 (C-3), 177.71 (C-4), 163.05 161.58 (C-5), 99.8 (C-6), 165.35 (C-7), 94.41 (C-8), 156.8 (C-9), 104.00 (C-10), 121.49 (C-1′), 115.61 (C-2′), 149.98 (C-3′), 147.41 (C-4′), 113.5 (C-5′), 122.7 (C-6′), 104.61 (C-1″), 74.16(C-2″), 77.12 (C-3″), 70.6(C-4″), 76.42 (C-5″), 67.37 (C-6″), 101.59 (C-1‴), 70.7 (C-2‴), 70.5 (C-3‴), 71.8 (C-4‴), 68.6 (C-5‴), 17.9 (C-6‴), 56.2 (O-Me). Accordingly, it was proposed to be isorhamnetin-3-*O*-*β*- rutinoside[18]. This compound was used as standard reference for the standardization of EFAM.

### 2.4. Standardization of EFAM Using Ultra Performance Liquid Chromatography (UPLC) Analysis

**Figure 1:**
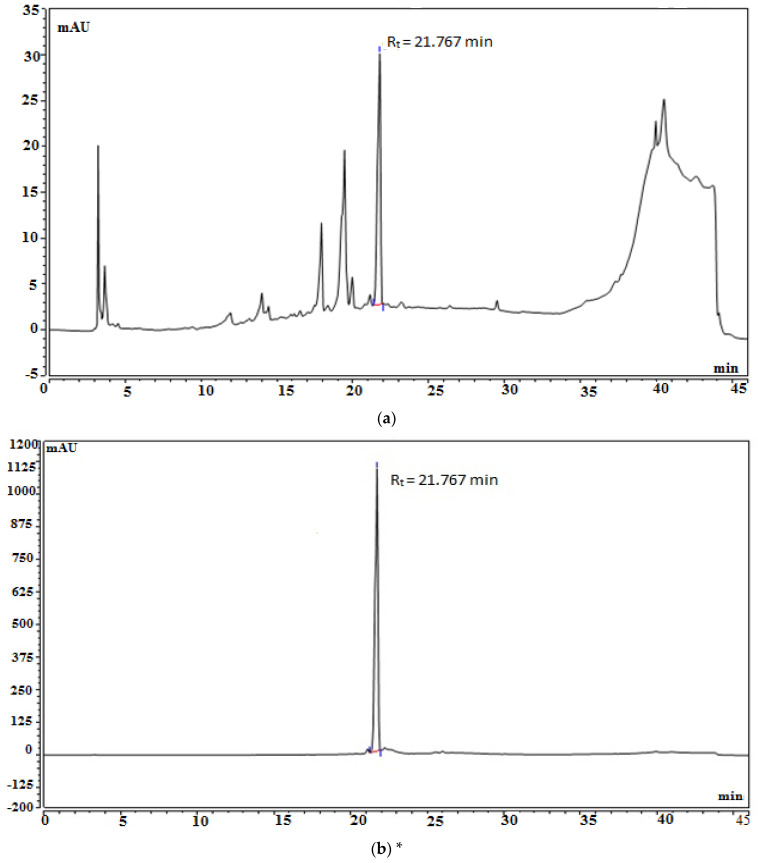

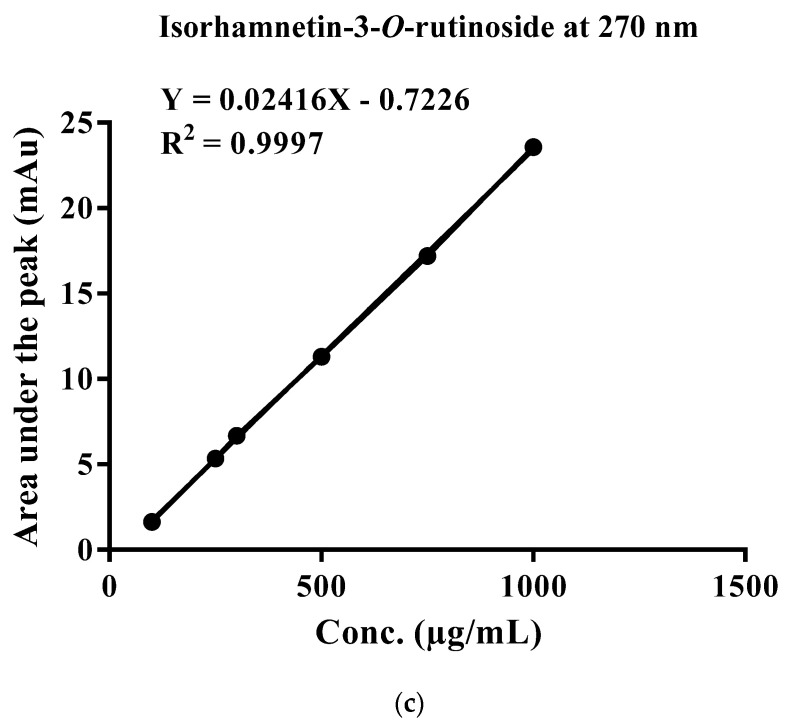
Standardization of EFAM: (**a**) HPLC Chromatogram of the ethyl acetate fraction; (**b**) * HPLC chromatogram of the isolated isorhamnetin-3-*O*-rutinoside (the purity of the isolated isorhamnetin-3-*O*-rutinoside is 99%); (**c**) calibration curve of the isolated isorhamnetin-3-*O*-rutinoside.

The PDA chromatogram of EFAM was recorded at 270 nm (Figure 1a) and showed a major peak (44.54%) at Rt = 21.7 min, corresponding to isorhamnetin-3-*O*-rutinoside. A calibration curve of the isolated isorhamnetin-3-*O*-rutinoside was established on the same UPLC device with the same conditions and parameters (Figure 1b). The calibration curve equation was found to be Y = 0.02416X − 0.7226, and the correlation coefficient was found to be R^2^ = 0.9997 (Figure 1c). Each 1 mg of EFAM was shown to constitute 262.91 ± 0.57 µg of isorhamnetin-3-*O*-rutinoside.

### 2.5. Characterization of EFAM Polyphenols by LC-MS/MS

This is the first time that the polyphenolic profile of EFAM has been reported utilizing UPLC-PDA-ESI-MS/MS (Table 2). Twenty-five compounds were tentatively identified, including phenolic acids and their derivatives (four compounds). The rest of the compounds were flavonoids and their derivatives.

Compound **1** showed a molecular ion peak at *m*/*z* 179 and a base peak in the MS/MS spectrum negative mode at *m*/*z* 135, corresponding to [M − H–CO_2_^−^]^−^, which was in agreement with the reported data on caffeic acid [19]. Compound **2** was identified as sinapic acid hexoside, with an [M − H]^−^ at *m*/*z* 385 and a base peak at *m*/*z* 223 [M − H–162]^−^, attributed to the loss of a hexosyl radical [19]. Compounds **3** and **6** were identified as caffeoyl-hexose-deoxyhexoside based on the [M − H]^−^ at *m*/*z* 487.0 and the fragment ion peak detected at *m*/*z* 308 by the expulsion of caffeic acid moiety at *m*/*z* 179 due to the loss of deoxyhexose and hexose moieties [20]. Compound **4** showed an [M − H]^−^ ion peak at *m*/*z* 431.1 with the fragment ion at *m*/*z* 299 owing to the loss of a pentoside moiety. This compound was identified as chrysoeriol pentoside [15]. Compound **5** exhibited a molecular ion peak at *m*/*z* 521.0. It yielded an anion at *m*/*z* 375 owing to the base peak and another fragment ion peak at *m*/*z* 331, indicating the breaking down and withdrawal of CO_2_ in the MS2 spectrum; the peak abundance of both ions was similar to that of biflavones in specific [21]. Thus, it could be potentially identified as a dihydroxy-amento flavone. Compounds **7** and **22** were suggested to be quercetin 3-*O*-*β*-D-glucoside based on the molecular ion peak at *m*/*z* 463. In the MS2 spectrum, an aglycone ion fragment peak at *m*/*z* 301 [M − H–162]^−^ was detected due to the removal of a hexosyl radical [22]. Compound **8** showed a major peak of [M − H]^−^ at *m*/*z* 447. Two ion fragments were distinguished in the MS2 spectrum, including a peak at *m*/*z* 285 (-162 Da) and a peak at *m*/*z* 284 (-163 Da) representing kaempferol aglycone, which was confirmed by the characteristic fragmentation pattern with an intense peak at *m*/*z* 151. The loss of 162 Da moiety revealed the presence of a hexoside moiety. The intensity of [M − H–162]^−^ at *m*/*z* 285 was characteristic of 3-*O*-glycosylation. The mass spectrum tentatively established compound 8 as kaempferol 3-*O*-*β*-D-glucoside [25]. Compound **9** showed a base peak at *m*/*z* 625.3 [M − H]^−^ and fragments at *m*/*z* 463 and 301, which indicated the sequential loss of hexosyl radicals, suggesting its identification as quercetin-3-*O*-diglucoside [27]. Compound **10** produced a precursor ion peak [M − H]^−^ at *m*/*z* 447.23 which produced a [M − H–146]^−^ ion at *m*/*z* 301, attributed to the cleavage of rhamnosyl radical. Thus, it was suggested to be quercetin-3-*O*-rhamnoside [22]. Similarly, compound **11** was referred to isorhamnetin-3-*O*-rhamnoside [28]. Compounds **12** and **15** showed a base peak at *m*/*z* 623.12 and a fragment ion peak at *m*/*z* 315, indicating that the aglycone (iso)rhamentin was left after the removal of the rutinose group (308 Da) as a base peak, indicating a (1–6) interglycosidic linkage [18]. Compound **13** showed a molecular ion peak [M − H]^−^ at *m*/*z* 769.18. A parent ion peak was produced [M − H]^−^ at *m*/*z* 454 owing to the removal of two rhamnose and hexose units and a fragment ion peak at *m*/*z* 315, indicating the presence of the aglycone isorhamentin; thus, it was identified as isorhamnetin-dirhamnopyranosyl-hexoside [18]. Compound **14** produced a deprotonated molecule at *m*/*z* 803.21 in which the fragmentation pattern showed ion peaks at *m*/*z* 477 because of isorhamnetin glucoside, which was further fragmented to the aglycone isorhamnetin product ion at *m*/*z* 315 by losing a glucose moiety. Moreover, the other characteristic fragment was *m*/*z* 623, corresponding to isorhamnetin-3-*O*-neohesperidoside; this was identified as isorhamnetin-3-*O*-glucosyl-neo-hesperidoside [15]. Compound **16** showed a base peak at *m*/*z* 577.3. The way these fragments were formed was identical to a di C-glycosyl flavone. It produced fragment ion peaks at *m*/*z* 503 [M − H–74]^−^ and *m*/*z* 473 due to [(M − H)–104]^−^, which are the characteristic peaks of a *C*-linked rhamonsyl. Other fragment ions at *m*/*z* 383 [M − H–90]^−^ and *m*/*z* 353 [M − H–120]^−^ were characteristic of a *C*-linked glucosyl. The intensity of the fragment ion peak produced from the carbon-linked glucosyl radical was higher than that produced from the carbon-linked rhamnosyl, which confirmed that the glucose moiety was fixed to the carbon at the sixth position and the rhamnose moiety was at the eighth position; therefore, the compound was referred to as apigenin-8-*C*-rhamnosyl-6-*C*-glucoside [30]. Furthermore, compound **17** showed an [M − H]^−^ peak at *m*/*z* 593.17 and a base peak at *m*/*z* 285 [M − H–308]^−^ owing to the loss of a rutinose moiety, and was suggested to be kaempferol-3-*O*-rutinoside [28]. Compound **18** was identified as isorhamnetin-3-*O*-glucoside, with a molecular ion peak at *m*/*z* 477.2 [M − H]^−^ and a base peak at *m*/*z* 315, attributed to the aglycone (iso)rhamnetin product after losing a hexosyl group (162 Da) [26]. Compound **19** was identified as 3′-*O*-methylorobol, with a [M − H]^−^ ion at *m*/*z* 299.12 and a base peak at *m*/*z* 284 [M − H–15]^−^ corresponding to losing a methyl radical [19]. The data on compounds **20** and **21** were in agreement with the reported data on the characteristic fragmentation pattern of quercetin and kaempferol aglycones [28,33]. Compound **23** was proposed to be 5,7-dihydroxy-4′-methoxy-6, 8-diprenylisoflavone, as it showed an [M − H]^−^ ion peak at *m*/*z* 419.08 together with fragment ion peaks at *m*/*z* 404 [M − H–15]^−^ and *m*/*z* 361 [M − H–15–43]^−^ owing to the sequential removal of methyl and an isopropyl radical [34]. Compound **24,** with [M − H]^−^ at *m*/*z* 315.2 and fragment ions at *m*/*z* 301 and 272, was referred to as (iso)rhamnetin aglycone [18].Compound **25** showed an [M − H]^−^ peak at *m*/*z* 315 and a base peak at *m*/*z* 300 [M − H–15]^−^, owing to the typical demethylation of tamarixetin [35].

### 2.6. In Vitro Assay

#### 2.6.1. DPPH Assay

The DPPH assay results (Table 3) showed that the EFAM exhibited the most potent antioxidant effect in comparison with the CEAM and BFAM, with IC_50_ (27.73 ± 1.85 µg/mL). The EFAM results were comparable to Trolox, the positive control.

#### 2.6.2. FRAP Assay

The FRAP assay results (Table 3) revealed that the EFAM showed the most potent antioxidant effect (176.60 ± 5.21 μM Trolox equivalent (TE)/mg fraction), followed by the CEAM and BFAM.

#### 2.6.3. Evaluation of the Cytotoxic Activity of the Different Fractions of *A. maurorum* (MTT Assay)

**Figure 2:**
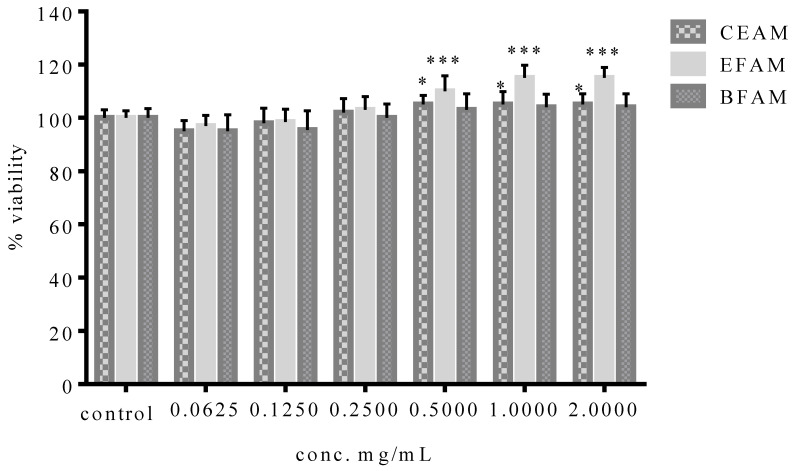
Viability of the oral epithelial cell OEC treated with CEAM, EFAM, and BFAM for 24 h. Data show the percentage of OEC viability at each concentration (*n* = 6). * *p* < 0.05, *** *p* < 0.001 using One-way ANOVA followed by a Schiff multiple range test. Results are shown as mean ± S.D.

Before assessing the antiulcer activity of the CEAM, EFAM, and BFAM, their cytotoxicity against the OEC cell line was evaluated by 3-(4,5-dimethylthiazolyl-2)-2,5-diphenyltetrazolium bromide (MTT) assay. For 24 h, OEC was subjected to CEAM, EFAM, and BFAM. Only the EFAM and CEAM exhibited statistically significant cytotoxic effects after 24 h at doses of 0.5 mg/mL or higher. According to these findings, EFAM, BFAM, and CEAM exhibited no effect on cell viability at concentrations equal to or greater than 0.5 mg/mL. On the other hand, EFAM induced cell proliferation starting from the treated concentration (0.5 mg/mL), leading to a 5.56 ± 2.52% increase in cell proliferation (*p* < 0.05) (Figure 2).

### 2.7. In Vivo Assay

#### 2.7.1. Histopathological Examination

**Figure 3:**
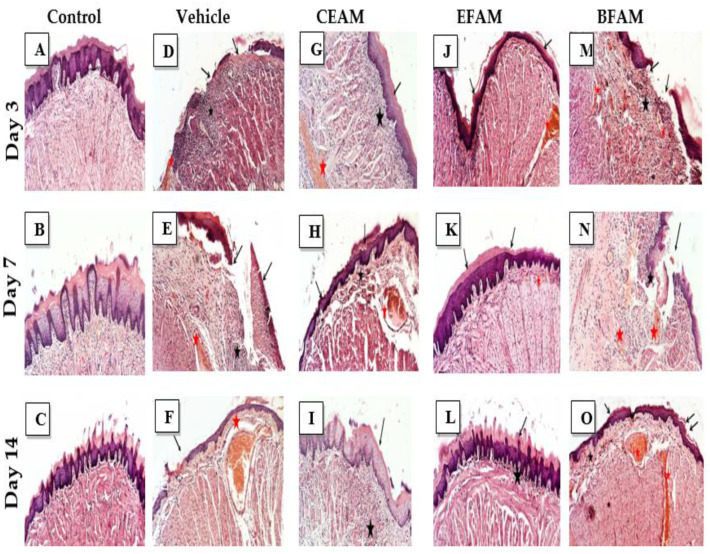
Photomicrographs of tongue sections stained with H and E (100×). Negative control rats (**A**–**C**), vehicle (**D**–**F**), CEAM (**G**–**I**), EFAM (**J**–**L**), BFAM (**M**–**O**) on days 3, 7 and 14, respectively.

The lingual mucosa in the control group showed normal histomorphological structure over 14 days (Figure 3A–C); however, the lingual mucosa in the vehicle-treated group showed minimal regeneration, as represented by the large focal necrotic and ulcerative lesions in the covering mucosa (*black arrow*) (Figure 3D). These features were accompanied by many constricted and dilated blood vessels (*red* star) and submucosal/intermuscular mononuclear and polymorphonuclear inflammatory cell infiltrates (*black star)* (Figure 3E,F). Upon treating the tissues with the CEAM, an apparent intact covering epithelium with mild focal degenerative changes in the basal cells layer (*arrow*) and focal areas of subepithelial hemorrhage together with inflammatory cells infiltrates (*black star*) along with many congested and dilated blood vessels (*red* star) were observed on day three after acetic acid treatment (Figure 3G). However, from day 7 to 14 an almost intact covering epithelium with a thin keratin layer appeared (*arrow*), together with moderate submucosal edema and inflammatory cells records (*black star*) and accompanied by large dilated and congested blood vessels (*red star*) (Figure 3H–I). The treatment with EFAM showed the most potent protective effect on day three, with a nearly intact covering of epithelium (*arrow*) as well as submucosal tissue with minimal inflammatory cell infiltrates. However, some dilated and congested submucosal blood vessels (*red* star) were observed (Figure 3J).

The EFAM markedly improved ulcer healing during the treatment period and helped to form a complete and thickened squamous epithelium with keratin layer (*arrow*); occasional mild congestion of submucosal blood vessels was observed (*red star*) (Figure 3K).

On day 14, an apparently intact and more keratinized covering epithelium (*arrow*) with mild focal submucosal inflammatory cells (*black star*) showed minimal abnormal alterations among the different groups (Figure 3L).

The BFAM-treated group showed focal ulcerative and hemorrhagic lesions on both the third and seventh days after acetic acid treatment (*black arrow*), with moderate submucosal inflammatory cells infiltrates (*black star*) and many congested blood vessels (*red star*) (Figure 3M–N). On the 14th day, moderate submucosal inflammatory cells and edema (*black star*) were observed together with an almost-intact covering epithelium with a thin keratin layer (*arrow*), accompanied with many dilated and congested lingual blood vessels (*red star*) (Figure 3O).

#### 2.7.2. Inflammatory Markers

**Figure 4:**
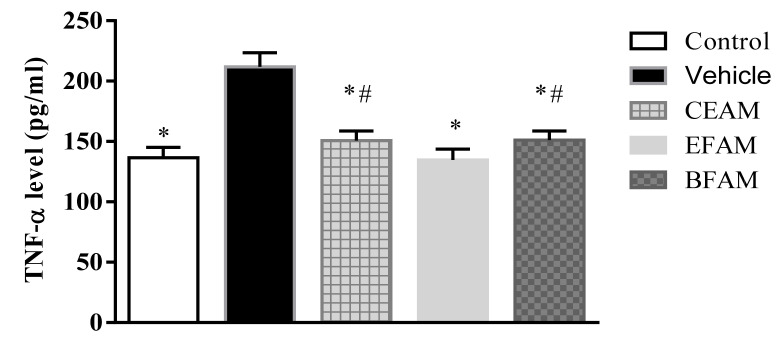
Effect of the *A. maurorum* extract and fractions on tongue tissue TNF-*α* level in acetic acid-induced tongue ulcers in rats compared with the vehicle and control rats. * *p* < 0.0001 vs. vehicle group and ^#^ *p* < 0.05 vs. EFAM group, using One-way ANOVA followed by Tukey–Kramer as a post hoc test. Results are shown as mean ± SD (*n* = 6).

Table 4 shows the effect of *A. maurorum* on the level of TNF-α in tongue tissues in the experimental group of rats. Inducing ulcers using acetic acid caused the TNF-α level to increase significantly, by 155% at *p* < 0.0001 in comparison with the control group, indicating that the animal models were reproduced successfully. The administration of EFAM, CEAM, and BFAM showed that TNF-α levels were significantly decreased, by 36.4%, 28.9%, and 28.6%, respectively, in comparison with the vehicle-treated group (Figure 4).

**Figure 5:**
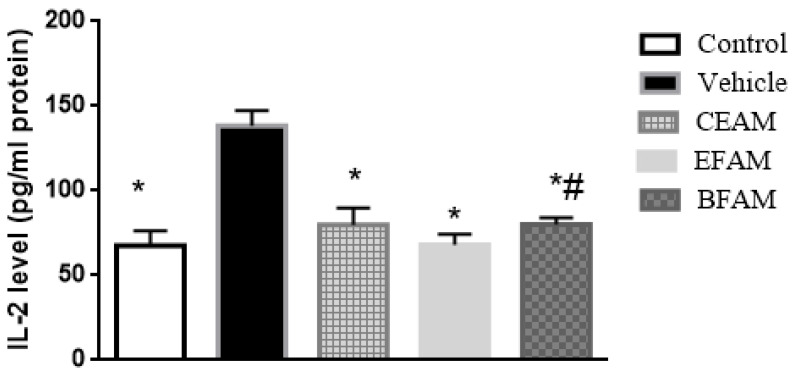
Effect of *A. maurorum* extract and fractions on tongue tissue IL-2 level in rats subjected to acetic acid-induced tongue ulcers compared with the vehicle group. * *p* < 0.05 vs. vehicle group and ^#^ *p* < 0.05 vs. EFAM group using One-way ANOVA followed by Tukey–Kramer as a post hoc test. Results are shown as mean ± SD (*n* = 6).

The effect on IL-2 levels was similar to that on TNF-α, as they were increased two-fold at *p* < 0.0001 upon exposure to acetic acid compared to the control group. The damaging effect of the ulcerative acetic acid was ameliorated by the administration of EFAM, CEAM, and BFAM, which reduced IL-2 levels by 50.8%, 42.3%, and 42.2%, respectively, in comparison with the vehicle-treated group (Figure 5).

#### 2.7.3. Immunohistochemical Staining of Proliferating Cell Nuclear Antigen (PCNA)

**Figure 6:**
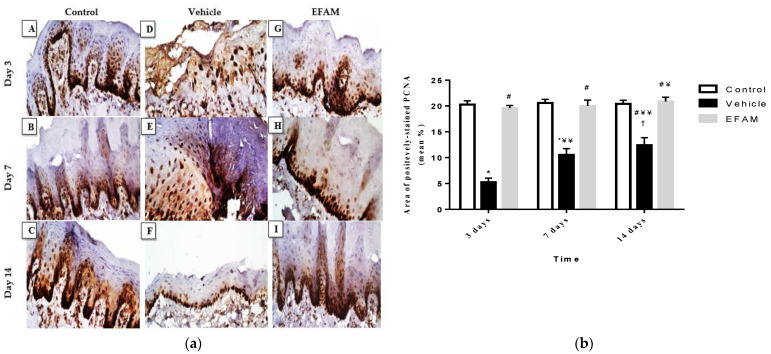
(**a**) Immunohistochemical staining of the positively-stained PCNA in the tongue ulcer (magnification, ×400, the scale bar indicates 50 µ). (**A**–**C**): Normal rats were treated with the vehicle only on days 3, 7, and 14, respectively. (**D**–**F**): Acetic acid-induced tongue ulcer rats treated with vehicle only on days 3, 7, and 14, respectively. (**G**–**I**): Acetic acid-induced tongue ulcer rats treated with EFAM on days 3, 7, and 14, respectively. The brown precipitate represents positively-stained PCNA. (**b**) Amount of positively immunostained PCNA, expressed as the mean area percentage (mean%), in the control, vehicle, and EFAM groups on 3, 7, and 14 days. * *p* < 0.0001 vs. control group of the same time interval, and # *p* < 0.0001 vs. vehicle group at the same time interval, ^¥^ *p* < 0.05, ^¥¥^ *p* < 0.0001 vs. respective group on day 3 and ^†^ *p* < 0.05 vs. respective group on day 7 using two-way ANOVA followed by Bonferroni as a post hoc test. Results shown as mean ± SD (*n* = 6).

Immunohistochemical positively-stained cells of PCNA were observed in the tongue ulcers; this reaction was localized on the epithelium owing to re-epithelialization and restoration of the tongue mucosa. Microscopical examination of PCNA immunohistochemistry revealed a faint brown stain in the vehicle-only treated group, while positive brown staining was highly expressed in the EFAM group over the 14 days of treatment (Figure 6A). In addition, there was a significant rise between the vehicle and EFAM groups on the 14th day (12.6 ± 1.9 and 21 ± 1.1; *p* < 0.0001) (Figure 6B). In the EFAM group, there was a significant rise in the area of positively-stained PCNA cells on day 14 when compared with the same respective group on day three (19.6 ± 0.6 and 21 ± 1.1; *p* < 0.05). These results demonstrated that there was an increase in the proliferation of cells in the healing regions of the lingual mucosa in EFAM topically-treated rats.

## 3. Discussion

Many natural secondary metabolites demonstrated various biological effects [37,38,39,40,41], including antioxidant potential via their ROS scavenging abilities [39,42,43]. The high antioxidant activity of polyphenols in oral mucosa is most likely responsible for their ability to protect tissues against periodontal and dental diseases, as polyphenols come into contact with oral tissues before being absorbed or digested [44,45]. Polyphenols reach the highest concentration on oral mucosa in comparison with all other tissues of the gastrointestinal tract [38]. Plant polyphenols and flavonoids also demonstrated anti-inflammatory properties in both in vitro and in vivo models [43,46,47]. These anti-inflammatory activities can be achieved through scavenging free radicals, regulating cellular activities in inflammatory cells, and modulating the activity of inflammatory enzymes such as phospholipase A2, COX, and NOS, as well as through modulating the expression of other proinflammatory cytokines such as IL-1β and TNF-α [46,48,49]. 

EFAM was found to possess the highest total phenolic (53.19 mg GAE/g dry extract) and flavonoid (47.96 mg RE/g dry extract) content, and showed both the most potent radical scavenging capacity against DPPH (IC_50_ = 27.73 ± 1.85 µg/mL) and the highest reducing power (a FRAP value of 176.60 ± 5.21 μM TE/mg extract). This encouraged us to standardize and highlight the polyphenol profile of EFAM and compare its oral antiulcer activity to those of BFAM and CEAM. The polyphenolic profile of EFAM was reported for the first time using the UPLC/PDA/ESI/MS method. Twenty-five polyphenol derivatives were tentatively identified. Most of the identified polyphenols in the EFAM were reported previously in *A. maurorum*, although caffeoyl-hexose-deoxyhexoside, dihydroxybiflavone derivative, (iso)rhamnetin rhamnoside, apigenin-rhamnosyl-hexoside, and dihydroxy methoxy diphenyl isoflavone were identified in the current study for the first time. Phytochemical investigation of EFAM resulted in the isolation and identification of two major flavonoids, isorhamnetin-3-*O*-rutinoside and quercetin-3-*O*-rhamnoside, both of which were previously reported in *A. maurorum* [18].

Accumulating evidence has shown that inflammatory reactions and reactive oxygen species contribute to the etiology of oral ulcer lesions [50], and that inflammation results in delayed wound healing. To achieve an effective antiulcer therapy, the inhibition of the proinflammatory cycle can be targeted [51]. These findings explain the activity of plants containing polyphenols in improving oral ulcers by reducing proinflammatory markers, scavenging free radicals, and decreasing oxidative stress [50,52].

The current study is the first to report the efficacy of different fractions of *A. maurorum* in healing tongue ulcers. Treatment with acetic acid resulted in severe histopathological destruction (necrosis, skin lesions, and inflammation) as well as an increase in the levels of the tongue tissue proinflammatory cytokines TNF-α and IL-2. Biochemical investigation proved that the topical application of EFAM in the treatment group significantly decreased inflammation, as demonstrated by the reduction in the levels of mucosal proinflammatory TNF-α and IL-2 after days 3, 7, and 14, with a gradual decrease from one period to another. This is in agreement with previous reports [53,54] finding that the improvement in healing of induced oral ulcers in in vivo studies was associated with a decrease in mucosal levels of proinflammatory cytokines. Pourahmad et al. [11] reported that *A. maurorum* as a source of flavanones could inhibit macrophage activity in oral lesions and thereby inhibit the expression of TNF-α and mediators of inflammation; this is in agreement with the present study, as EFAM improved the healing of aphthous ulcers in comparison with the vehicle-only treatment group.

These effects were further confirmed by histopathological investigation of the CEAM and EFAM groups, which showed minimal damaging effects as represented in the gradual reduction in inflammation, restoration of mucosal epithelium, and appearance of a keratin layer all occurring gradually over the 14 days of treatment. General improvement in the pathological structure of the acetic acid induced-tongue ulcers was observed compared to the vehicle-only treated rats, which showed signs of epithelial atrophy, absence of a keratin layer and the presence of inflammatory cells infiltration. The best healing effect was found in the EFAM-treated group at the end of the 14-days treatment period. Pourali and Yahyaei [55] studied the role of *A. maurorum* in enhancing wound healing through tissue remodeling and collagen deposition, which is in agreement with the results of the present study. Ulcer healing is a complex programmed process that requires epithelial cell migration and proliferation for continued re-epithelialization [56]. PCNA shows a vital role in proliferating and repairing DNA in the cells. Treatment with EFAM increased the number of PCNA-positive cells localized in the tongue mucosa. This indicates that one of the mechanisms of *A. maurorum* in accelerating ulcer healing is probably through increased cell proliferation. Structural analysis of the flavonoids showed that the double bond between carbon 2 and carbon 3, the hydroxyl groups at 5, 7, 3′, and 4′, and the position of ring B at carbon 2 strongly suppressed cytokine expression [57]. It was proven that isorhamnetin rutinoside could potentially inhibit expression of pro-inflammatory mediators (TNF-α and IL-6), explaining its anti-inflammatory activity [58]. Isorhamnetin rutinoside has been evaluated for its local anti-inflammatory property in croton oil-induced rat ear edema, with a strong impact on TNF-α expression resulting in 83.3% reduction [59]. Moreover, many reports have proven that glycosylated flavonoids are potent inhibitors of cytokine expression [60,61]. A recently isolated new isorhamnetin glycoside derivative (isorhamnetin-3-O-[2′,3′-*O*-isopropylidenea-L-rhamnopyranosyl] -(1′-6′)-O-b-D-glucopyranoside) exhibited anti-inflammatory effect with potent inhibition of the mRNA expression of IL-6 and TNF-α[58]. Quercetin-3-*O*-rhamnoside was reported to inhibit the primary vascular response mediated by amines and prostaglandins, and produced a reduction of 47% in the level of myeloperoxidase activity on a mouse ear edema model [62]. In Salaverry et al. [63] it was shown that aqueous extract of *Smilax campestris* with quercetin-3-*O*-rhamnoside as the main component significantly reduced production of pro-inflammatory cytokines along with tumor necrosis factor (TNF)-α, interleukin (IL)-1β, IL-6, IL-8 and monocyte chemoattractant protein (MCP)-1, and reduced the activity of metalloproteinase (MMP)-9 in lipopolysaccharide-activated macrophages as well as.

## 4. Materials and Methods

### 4.1. Materials

The aerial parts of *A. maurorum* Boiss, F. Leguminosae were obtained from Aswan, Egypt. Authentication was achieved by Therese Labib, Consultant at El-Orman Botanic Garden, Giza, Egypt. A voucher specimen was deposited at the herbarium (PHG-P-AM-243) at the Department of Pharmacognosy, Faculty of Pharmacy, Ain Shams University, Egypt. All solvents were of analytical grade, while those used in the preparative HPLC and UPLC/PDA/ESI/MS assays were of HPLC grade. Rutin, gallic acid, and Trolox were obtained from Sigma-Aldrich (Schnelldorf, Germany). ELISA kits, including TNF-α kit Catalogue No. CSB-E1252r, were purchased from Cusabio (Huston, TX, USA), and the IL-2 kit Catalogue No. E-EL-R0027 was purchased from Elabscience (Huston, TX, USA).

### 4.2. Plant Extraction and Fractionation of A. maurorum

Air-dried powdered aerial parts of *A. maurorum* (1 kg) were thoroughly extracted by maceration with 70% ethanol (1 L × 3) for 48 h at room temperature followed by filtration. The extracts were dried at 45 °C using a rotary vacuum evaporator, yielding 95 g (9.5%). For further analysis, 15 g of the dried hydro-alcohol extract was labeled as CEAM and freeze-dried. The remaining dried extract was dissolved in 400 mL of distilled water. The aqueous solution was fractionated using increasing polarity solvents such as *n*-hexane, ethyl acetate, and *n*-butanol. The dried fractions yielded 20, 18, and 23 g, respectively. The CEAM (15 g), EFAM (18 g), and BFAM (23 g) were freeze-dried for phytochemical and biological investigation. The CEAM, EFAM, and BFAM were subjected to further investigation, while the *n*-hexane fraction was prepared as a first step in purification in order to extract the nonpolar compounds not belonging to the class of polyphenols.

### 4.3. Determination of Total Polyphenolic and Flavonoid Content of A. maurorum

The total polyphenolic and flavonoid content of the CEAM, EFAM, and BFAM was assayed using the micro-plate adapted Folin–Ciocalteu and aluminum chloride methods, respectively, using Fluostar Omega microplate reader (BMG Labtech, Ortenberg, Germany) [64,65]. Gallic acid and rutin were employed as the standards to determine the total polyphenolic content (mg GAE/g extract or fraction) and flavonoid content (mg RE/g extract or fraction), respectively. For the calibration curve establishment, gallic acid concentrations ranged from 7.8 to 500 g/mL, while rutin concentrations ranged from 10 to 1000 g/mL.

### 4.4. Isolation and Identification of the Major Polyphenolic Compound of EFAM

The isolation of the major compounds in the EFAM was carried out by High Performance Liquid Chromatography technique using a Waters 2695 Alliance HPLC system with a Waters photodiode array detector equipped with preparative column (C_18_ Kromasil 1 cm × 250 mm, 5 µm) (Waters Corp., Milford, MA, USA). For system control and data acquisition, Empower 3.0 Software (Waters, Milford, MA, USA) was used. The sample solvent was methanol with a concentration of 1 mg/mL; filtration using a 0.45 µm syringe filter was then carried out. The mobile phase system consisted of two phases: (A) 0.1% formic acid in water, and (B) 0.1% formic acid in methanol, with the following gradients: 0–3 min (10% B), 3–25 min (10–70% B), 25–30 min (70% B), 30–35 min (70–100% B), 35–40 min (100% B), 40–41 min (100–10% B), 41–46 min (10% B), monitored using PDA (190 to 800 nm). The flow rate was 3 mL/min and the injection volume was 100 μL. The structures of the isolated compounds were elucidated by ^1^H and ^13^C NMR spectroscopical analyses conducted on Bruker Avance III HD 400 MHz (Bruker AG, Fällanden Switzerland) [19].

### 4.5. Standardization of EFAM Using Ultra Performance Liquid Chromatography (UPLC) Analysis

The polyphenol-rich EFAM fraction was standardized. The experiment was carried out on a Thermo Fisher UPLC Model Ultimate 3000 (Agilent, Santa Clara, CA, USA) equipped with PDA–UV–Visible light detector, a Hypersil GOLD column (250 mm × 4.6 mm i.d.) and particle size 5 µm. A calibration curve of the isolated main flavonoid isorhamnetin-3-*O*-rutinoside was established with a concentration range of 100–1000 μg/mL; the EFAM concentration was 1 mg/mL. All experiments were performed in three replicates [66].

### 4.6. UPLC/PDA/ESI/MS/MS Analysis of Polyphenol-Rich Fraction (EFAM)

The profiling of the chemical constituents was performed according to [67] using mass spectrometric analysis carried out on a Waters™ ACQUITY Xevo™ TQD system composed of an ACQUITY UPLC H-Class system and a XevoTMTQD triple-quadrupole tandem mass spectrometer ESI (-ve mode) as the electrospray ionization (ESI) interface (Waters Corp., Milford, MA, USA). The column used was a C_18_ 100 mm × 2.1 mm column (p.s., 1.7 µm) (Waters, Ireland). The sample solvent was methanol at a concentration of 1 mg/mL, and the sample was filtered using a 0.2 µm micropore filter. The mobile phase system consisted of two phases: (A) 0.1% formic acid in water, and (B) 0.1% formic acid in acetonitrile, with the following gradients: 0–4 min, 15% B; 4–8 min, 20% B; 8–30 min, 55% B; 30–35 min, 90% B; 35–40 min, 15% B, with a flow rate of 200 μL/min and the injection volume adjusted at 10 µL. The scan range was 100–1000 m/z. The following settings were applied to the instrument: capillary voltage 3.5 kV; detection at cone voltages 20 V–95 V; radio frequency (RF) lens voltage 2.5 V; source temperature 150 °C; desolvation gas temperature 500 °C. Nitrogen was used as desolvation and cone gas at a flow rate of 1000 and 20 L/h, respectively. MassLynx 4.1 software (Waters, Milford, MA, USA) was used to control system operation and for data collection. 

### 4.7. In Vitro Studies

#### 4.7.1. DPPH Assay

DPPH free radical assays of CEAM, EFAM, and BFAM were performed according to [68]. All assays were carried out in three replicates. Trolox was used as the positive control.

#### 4.7.2. FRAP Assay

The antioxidant power of CEAM, EFAM, and BFAM was determined by FRAP assay using a FluoStar^®^ Omega microplate reader (BMG LABTECH, Ortenberg, Germany). Trolox was employed as the standard, with concentrations ranging from 50 to 4000 μM to calculate the antioxidant power presented as μM TE/mg extract or fraction [69].

#### 4.7.3. Cytotoxic Activity (MTT Assay)

Oral epithelial cell OEC cell line was cultivated as adherent cultures on Petri dishes and kept at 37 °C, as reported by Picerno et al. [70]. The MTT reduction test was used to assess cell viability according to Lopes et al. [71]. The cell viability findings are presented as the mean ± SD of not less than four separate assays carried out in three replicates, and are presented as the percentage of the untreated control cells.

### 4.8. In Vivo Traumatic Ulcer Healing Study

#### 4.8.1. Animals

Adult male Sprague Dawley rats (150 and 200 g) were obtained from the Agricultural Research Center, Giza, Egypt. The rats were housed in separate cages, three rats per cage in the animal house facility of the Faculty of Pharmacy, Ain Shams University on a 12 h light–dark cycle in a temperature- and humidity-controlled room (21–23 °C and 40–60%, respectively). They were fed on standard diet pellets for rodents (El Nasr, Egypt) and given water ad libitum. All animal handling steps were performed following the rules of the Ain Shams University Faculty of Pharmacy Ethical Committee for the use of animal subjects (No. 27, March 2020).

#### 4.8.2. Induction of Tongue Ulcers

All rats were given pentobarbital intraperitoneal as anesthesia in a dose of 50 mg/kg. Tongue ulcers were chemically induced in all rats except the control groups using cotton swabs soaked in 20 µL of 50% acetic acid pressed onto the dorsal surface of the tongue for 1 min [72].

#### 4.8.3. Treatment Preparation

The topical gel was formulated as 5% of CEAM, EFAM and BFAM dispersed in 0.5% carboxymethyl cellulose. The extract and fractions were incorporated in the plain polymeric gel base using geometric addition in order to ensure uniform drug distribution throughout the base [72]. 

#### 4.8.4. Experimental Design

The rats were randomly allocated into five groups (*n* = 18) and were given the following treatment for 14 days. Group 1 (Control): rats were treated with 1 drop of the respective vehicle twice daily. Group 2 (Vehicle): rats underwent ulcer induction using acetic acid and were given 1 drop of topical treatment containing vehicle only twice daily. Group 3 (CEAM): rats underwent ulcer induction using acetic acid and were treated with 1 drop of a topical gel containing 5% CEAM twice daily. Group 4 (EFAM): rats underwent ulcer induction using acetic acid and were treated with 1 drop of a topical gel containing 5% EFAM twice daily. Group 5 (BFAM): rats underwent ulcer induction using acetic acid and were treated with 1drop topical gel containing 5% BFAM twice daily. Rats were prohibited from food and water for half an hour after each topical treatment application in order to assure its adhesion on the rats’ tongues [7].

On days 3, 7, and 14, six rats from each group were sacrificed with an overdose of pentobarbital. The tongues were dissected, weighed, and washed using ice-cold phosphate buffered saline (PBS) (pH 7.2). Specimens were taken from the tongues and prepared for biochemical, histopathological, and immunohistochemical analysis.

#### 4.8.5. Biochemical Assessment of Inflammatory Markers

Tongue specimens were frozen, washed in ice-cold PBS (pH 7.2), and ground to obtain 20% homogenate using a tissue homogenizer (mini-BeadBeater-8, BioSpec products, Bartlesville, OK, USA). Tissue fragments were then incubated in lysis buffer solution, placed on ice, sonicated for 30 sec, and centrifuged at 4000 rpm for 15 min using a Sigma 2-7 Centrifuge (Osterode am Harz, Germany). Then, the supernatant was collected and maintained at −80 °C until performing the ELISA assay. The TNF-α and IL-2 levels in tongue homogenates were measured using a commercial ready-made ELISA kit (Sigma Aldrich Chemical Co., St Louis, MO, USA) according to the manufacturer’s instructions. TNF-α and IL-2 contents were measured, presented as pg/mg protein. The protein concentration was determined using bovine serum albumin as the standard according to the Lowry method [73].

#### 4.8.6. Tongue Tissue Preparation for Staining

Specimens from the tongues were fixed in neutral buffered formalin (10%) for 72 h for histopathological and immunohistochemical analysis. The fixed tongue tissues were sequentially processed by alcohol and xylene and embedded in paraplast tissue-embedding medium. A rotatory microtome was used to cut serial 4 μm tissue sections. Each section was stained with Harris Hematoxylin and Eosin (H&E) as a conventional tissue examination staining method according to [74].

For the immunohistochemical staining, specimen paraffin blocks were cut by microtome at 4 μm thickness. The samples were deparaffinized and treated with 3% H_2_O_2_ for 15 min. The slides were incubated with the primary antibody anti-PCNA (MA5-11358, Thermo Fisher Scientific, Fremont, CA, USA) (1:100) for 1 h and washed using phosphate buffered saline (PBS). The cells were incubated with HRP-conjugated secondary antibody (EnVision-HRP kit, Agilent Dako, Santa Clara, CA, USA) for 20 min, rinsed with PBS, and treated with DAB chromogen for 10 min, then rinsed with PBS. The slides were counter-stained with Hematoxylin stain, dried, and cleared in xylene for microscopic examination and quantification [75].

#### 4.8.7. Histomorphometric Analysis

Six random non-overlapping fields per sample were selected for each positive anti-PCNA section, and photomicrographs were captured at ×400 magnification.

Morphometric analysis was performed by calculating the mean area percentage (mean%) of the brown-colored positively immunostained PCNA in the lingual covering mucosa, as per [76]. This was done by a specialist blinded to the experimental groups using image analysis software (Image J, 1.41a, NIH, USA). All photomicrographs were taken with a Leica Application Module linked to Full HD microscopic imaging equipment (Leica Microsystems GmbH, Wetzlar, Germany).

### 4.9. Statistical Analysis

Results are reported as mean ± standard deviation (SD), and were analyzed via one-way analysis of variance (ANOVA) followed by the Tukey–Kramer test for post hoc analysis in order to compare between different groups at the same time interval, and via two-way ANOVA followed by the Bonferroni post hoc test to compare between the groups at different time intervals. The cell viability assay findings were examined using one-way ANOVA followed by the Schiff multiple test. Statistical significance was defined as *p* < 0.05 probability values. All statistical analyses and graphs were performed and sketched using GraphPad Prism version 6.00 for Windows, GraphPad Software, La Jolla, CA, USA.

## 5. Conclusions

*Alhagi maurorum* ethyl acetate fraction, being a rich source of polyphenols, accelerated the healing process in tongue ulcers owing to its high antioxidant capacity, as represented by its potent free radical scavenging activity against DPPH, FRAP, its anti-inflammatory activity via decreasing the mucosal content of the proinflammatory cytokines TNF-α and IL-2, and its promotion of cell proliferation by increasing the expression of PCNA in ulcerative lesions. These findings suggested that *A. maurorum* is a good candidate for oral ulcer treatment, and warrants further clinical studies.

## Figures and Tables

**Table 1:** Total phenolic content (TPC as mg GAE/g dry extract) and total flavonoid content (TFC as mg RE/g dry extract) of different fractions of *A. maurorum*.

	Total Phenolic Content (mg GAE/g Dry Extract)	Total Flavonoid Content (mg RE/g Dry Extract)
CEAM	36.50 ± 2.2	22.90 ± 1.07
EFAM	53.19 ± 4.2	47.96 ± 3.1
BFAM	11.81 ± 0.36	4.21 ± 0.4

Data are expressed as mean ± SD, (*n* = 3).

**Table 2:** Peak assignment of the metabolites detected in the ethyl acetate fraction of *A. maurorum* using UPLC-PDA-ESI-MS/MS (negative mode).

Peak #	Rt (Min)	Identified Compound	UV-Vis (λmax)	[M − H]^−^ *(m*/*z)*	Fragment Ions *(m*/*z)*	Percentage (%)	Occurrence	Reference
1	2.5	Caffeic acid	---	179	135	0.2	*A. maurorum*	[19]
2	3.5	Sinapic acid hexoside	228	385.0	223, 179	0.65	*A. maurorum*	[19]
3	3.97	Caffeoyl-hexose-deoxyhexoside	318	487.0	308,179	6.40	-	[20]
4	4.5	Chrysoeriolpentoside (chrysoeriol-7-O-xylosoid)	220, 335	431.1	299	0.21	*A. maurorum*	[15]
5	7.85	Dihydroxybiflavone derivative	220, 332	521.01	375, 331	0.15	-	[21]
6	17.5	Caffeoyl-hexose-deoxyhexoside	320	487.0	308, 179	0.70	-	[20]
7	20.25	*Quercetin hexoside* *(*Quercetin3-O-*β*-D-glucoside)	221, 330	463	301, 179	1.56	*A. maurorum* *A. maurorum*	[22,23,24]
8	21.9	Kampferol hexoside (Kampferol-*3-O-β-D*-glucoside)	220, 330	447.02	285, 284	2.15	*A. maurorum* *A. sparsifolia*	[24,25,26]
9	22.12	Quercetin diglucoside (Quercetin-*3-O-di*-glucoside)	257, 335	625.3	271, 301,179	1.75	* A. maurorum *	[27]
10	22.77	Quercetin rhamnoside (quercetin-3-*O*-*β*- rhamnoside)	225, 332	447.23	301,179	3.85	*A. maurorum* *A. persarum*	[22,23,24]
11	23.7	(Iso)rhamnetin rhamnoside	235, 335	461	315	0.99	-	[28]
12	24.58	Isorhamnetin-3-O-rhamnopyranosyl hexoside (Isorhamnetin-3-*O*-*β*-rutinoside)	250, 342	623.12	315, 301, 179, 151	5.35	*A. maurorum* *A. maurorum*	[18,24,28]
13	26.5	(Iso)rhamnetin-dirhamnopyranosyl- hexoside Isorhamnetin-3-*O*-rhamnosyl-rutinoside (Typhaneoside)	230, 330	769.18	454, 315	0.63	* A. maurorum *	[18,29]
14	29.2	(Iso)rhamnetin 3-O-hexosyl-neo-hesperidoside (Isorhamnetin 3-O-glucosylneo-hesperidoside)	250, 350	803.21	623, 477, 315	0.75	*A. maurorum*	[15]
15	32.5	Isorhamnetin-3-O-rhamnopyranosyl hexoside (Isorhamnetin-3-*O*-*β*-rutinoside)	268, 342	623.21	315, 301, 179, 151	0.88	* A. maurorum* *A. maurorum *	[18,24,28]
16	34.47	Apigenin-rhamnosyl-hexoside (Apigenin-8-*C*-rhamnosyl-6-*C*-glucoside)	272, 373	577.3	503, 473, 383, 353	2.98	-	[30]
17	35.0	Kaempferol-3-*O*-rutinoside	262, 362	593.17	285, 284	0.42	*A. maurorum*	[28,31]
18	35.5	(Iso)rhamnetinhexoside (Isorhamnetin-3- *O*-*β*-D-glucopyranoside)	268, 342	477.2	315	0.48	*A. saparsifolia*	[26]
19	36.12	3′-O-methylorobol	283,357	299.12	284, 271	0.58	*A. maurorum*	[23,32]
20	36.8	Quercetin	254, 374	301.08	179, 151	0.83	* A. maurorum* *A. saparsifolia *	[18,26,28]
21	37.0	Kaempferol	222, 335	285.9	239, 187	0.85	*A. maurorum* *A. saparsifolia *	[18,26,33]
22	37.80	*Quercetin hexoside* *(*Quercetin-3-*O*-*β*-D-glucopyranoside)	221, 330	463	301, 179	2.35	*A. maurorum* *A. maurorum*	[22,23,24]
23	38.98	Dihydroxy methoxy diprenyl isoflavone (5,7-dihydroxy-4′-methoxy-6, 8-dipenylisoflavone)	225, 335	419.08	404, 361	4.50	-	[34]
24	39.33	Isorhamnetin	224, 338	315.2	301, 272	2.75	*A. maurorum*	[18,29]
25	40.5	Tamarixetin	220, 331	315	300, 151	3.12	*A. maurorum*	[35,36]

**Table 3:** Antioxidant activity of the different fractions of *A. maurorum* using DPPH and FRAP assays.

	DPPH IC_50_ (μg/mL)	FRAP (μM TE/mg Extract)
CEAM	45.22 ± 2.11	139.90 ± 4.01
EFAM	27.73 ± 1.85	176.60 ± 5.21
BFAM	52.48 ± 35.85	78.04 ± 7.92
Trolox	22.35 ± 0.87	-

Data are expressed as mean ± SD, (*n* = 3). Trolox was used as the positive control.

**Table 4:** Effect of the *A. maurorum* extract (CEAM), ethyl acetate (EFAM), and butanol (BFAM) fractions on tongue TNF-α and IL-2 levels in acetic acid-induced tongue ulcers in rats.

Group	TNF-α (pg/mL Protein)	IL-2 (pg/mL Protein)
Control	136.64 * ± 8.37	67.63 * ± 8.64
Vehicle	211.79 ± 11.69	138.33 ± 8.87
CEAM	150.65 *,# ± 8.08	79.87 * ± 9.84
EFAM	134.62 * ± 9.08	68.07 * ± 6.22
BFAM	151.16 *,# ± 7.47	80.02 *,# ± 4.12

Data are presented as the mean ± SD (*n* = 6). * or #: Significantly different from the vehicle or ethyl acetate fraction group at *p* < 0.05 using One-way ANOVA followed by Tukey–Kramer as a post hoc test.

## Data Availability

The data that support the findings of this study are openly available from the corresponding authors upon reasonable request.
